# Garadacimab for the long‐term prophylaxis of hereditary angioedema

**DOI:** 10.1111/ddg.70276

**Published:** 2026-04-30

**Authors:** Emel Aygören‐Pürsün, Regina Treudler, Petra Staubach, Inmaculada Martinez Saguer, Thomas Linhoff, Markus Magerl

**Affiliations:** ^1^ Goethe University Frankfurt Department of Pediatrics Frankfurt Germany; ^2^ Institute of Allergology Charité – Universitätsmedizin Berlin Corporate Member of Freie Universität Berlin and Humboldt‐Universität zu Berlin Berlin Germany; ^3^ Department of Dermatology University Medical Center Mainz Germany; ^4^ HZRM Haemophilia Centre Rhein Main Frankfurt Germany; ^5^ CSL Behring Hattersheim Germany; ^6^ Institute of Allergology Charité – Universitätsmedizin Berlin Corporate Member of Freie Universität Berlin and Humboldt‐Universität zu Berlin Berlin and Fraunhofer Institute for Translational Medicine and Pharmacology ITMP Immunology and Allergology Berlin Germany

**Keywords:** hereditary angioedema, FXIIa, garadacimab, long‐term prophylaxis

## Abstract

Hereditary angioedema (HAE), a rare and debilitating disease characterized by recurrent and spontaneous attacks of tissue swelling, has a high unmet therapeutic need, with many patients experiencing insufficient disease control with current prophylactic treatments. The goal of HAE treatment as per guidelines is to achieve total disease control and to normalize patients’ lives. Factor XII (FXII), the principal initiator of the contact system, ultimately regulates the kallikrein–kinin system. Activated FXII (FXIIa) converts plasma prekallikrein to plasma kallikrein, which cleaves high‐molecular‐weight kininogen to produce the vasoactive peptide bradykinin. In HAE pathophysiology, kallikrein–kinin system dysregulation results in excessive bradykinin production. The anti‐FXIIa monoclonal antibody garadacimab is approved for long‐term prophylaxis against HAE attacks. In the pivotal phase III (VANGUARD) and ongoing phase III open‐label extension studies, garadacimab administered subcutaneously (2 × 200 mg loading dose followed by 200 mg once monthly) demonstrated durable efficacy with early onset of protection and a favorable long‐term safety profile. Based on this and earlier clinical data, garadacimab has the potential to bring patients closer to achieving the guideline‐recommended goals of disease control and normalization of life. Here, we provide an overview of the results from the garadacimab clinical development program.

## INTRODUCTION

Hereditary angioedema (HAE) is a rare, potentially life‐threatening disease characterized by recurrent and spontaneous attacks of disfiguring and painful tissue swelling, mainly affecting the skin, abdomen, and upper respiratory tract.^1^, ^2^ As well as physical debilitation, patients experience decreased health‐related quality of life (HRQoL), with attacks impacting daily activities, work, and mental health, and ultimately requiring lifestyle modifications.^3^, ^4^, ^5^

**FIGURE 1:**
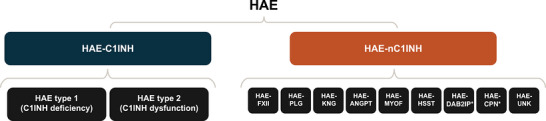
The role of the kallikrein–kinin system in HAE pathophysiology.^2^, ^6^, ^8^, ^15^, ^17^, ^70^, ^71^ *Abbr*.: ANGPT, angiopoietin‐1; C1INH, C1‐esterase inhibitor; CPN, carboxypeptidase N; DAB2IP, disabled homolog 2‐interacting protein; FXII, factor XII; *possible HAE‐nC1INH; HAE, hereditary angioedema; HAE‐C1INH, hereditary angioedema with C1‐esterase inhibitor deficiency or dysfunction; HAE‐nC1INH, hereditary angioedema with normal C1‐esterase inhibitor; HSST, heparan sulfate glucosamine 3‐O‐sulfotransferase 6; KNG, kininogen 1; MYOF, myoferlin; PLG, plasminogen; UNK, unknown origin.

HAE is an autosomal dominant genetic disorder with genetic/clinical heterogenicity; HAE types are classified according to the level and functional activity of the C1‐esterase inhibitor (C1INH) (Figure 1).^2^, ^6^ HAE with C1‐esterase inhibitor deficiency or dysfunction (HAE‐C1INH), which is the most common type, is caused by *SERPING1* genetic variants leading to either deficiency (HAE type 1) or dysfunction (HAE type 2) of C1INH, subsequently leading to dysregulation of the kallikrein–kinin system and overproduction of bradykinin.^2^, ^6^, ^7^, ^8^ HAE with normal levels of C1INH (HAE‐nC1INH) is a less common type of HAE caused by gene mutations in other proteins that may be involved in the formation or regulation of bradykinin.^2^, ^9^, ^10^

**FIGURE 2:**
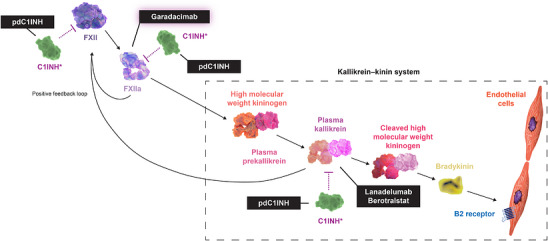
Depiction of the kallikrein–kinin system.^11^, ^12^, ^27^ *Intrinsic C1INH under normal physiologic conditions. *Abbr*.: C1INH, C1‐esterase inhibitor; FXII, factor XII; FXIIa, activated factor XII; pdC1INH, plasma‐derived C1‐esterase inhibitor

Factor XII (FXII) is the principal initiator of the contact system and ultimately regulates the kallikrein–kinin system (Figure 2).^6^, ^7^, ^11^, ^12^ The kallikrein–kinin system is a proteolytic cascade that leads to the production of the two proinflammatory mediators responsible for the pathophysiology of angioedema, plasma kallikrein, and bradykinin.^12^, ^13^, ^14^ Activation of the kallikrein–kinin system is initiated via contact activation, in which FXII undergoes a conformational change upon binding to negatively charged surfaces. This interaction results in the generation of the activated serine protease activated factor XII (FXIIa).^12^, ^14^ FXIIa converts plasma prekallikrein to its active form, plasma kallikrein, which cleaves high‐molecular‐weight kininogen to produce bradykinin.^7^, ^12^ Upon binding to the bradykinin receptor B2, bradykinin transiently mediates endothelial permeability, leading to vascular leakage and flow of water, ions, and proteins.^7^, ^12^, ^14^, ^15^ Together, FXIIa and plasma kallikrein generate a positive feedback loop by cleaving FXII, generating more FXIIa and amplifying bradykinin production.^12^, ^14^

Under normal physiologic conditions, C1INH (a potent serine protease inhibitor) tightly regulates bradykinin production in the kallikrein–kinin system by inhibiting FXIIa and plasma kallikrein and disrupting the positive feedback loop.^7^, ^16^ In patients with HAE‐C1INH, however, this system is dysregulated, which leads to an overproduction of bradykinin.^12^, ^17^ This results in excessive endothelial permeability and the swellings associated with an HAE attack.^12^, ^15^

## CURRENT HAE GUIDELINES

The goals of HAE treatment recommended by the *World Allergy Organization* (WAO)/*European Academy of Allergy and Clinical Immunology* (EAACI) and the *United States Hereditary Angioedema Association* (US HAEA) Medical Advisory Board are to achieve total disease control and to normalize patients’ lives by reducing or eliminating its interference with daily activities.^2^, ^6^ No longer experiencing HAE attacks is an important prerequisite for total disease control.^2^ The WAO/EAACI guidelines recommend on‐demand treatment to treat HAE attacks, short‐term prophylaxis to minimize the risk of attacks in advance of triggering events such as surgical or dental procedures, and long‐term prophylaxis (LTP) for the prevention of attacks.^2^ The WAO/EAACI guidelines also state that LTP is the only treatment strategy that can currently achieve total disease control and normalization of life and that the potential benefit of switching treatment to an LTP should be evaluated for patients at every clinical visit.^2^ The recommended first‐line treatments for LTP (Table 1) include C1INH therapy in the form of plasma‐derived C1INH (pdC1INH).^2^ C1INH therapy is intended to replace deficient or dysfunctional C1INH, thereby regulating kallikrein and bradykinin production (Figure 2).^2^ pdC1INH is approved as an LTP in the USA as Cinryze^®^ (intravenous [IV]) or HAEGARDA^®^ (subcutaneous [SC]) and in Europe as Cinryze^®^ (IV) or Berinert^®^ 2000/3000 (SC).^18^, ^19^, ^20^, ^21^ Other recommended first‐line LTP treatment options include lanadelumab (Takhzyro^®^; SC), a fully human monoclonal antibody against plasma kallikrein, and berotralstat (Orladeyo^®^; oral), a selective inhibitor of plasma kallikrein (Figure 2).^2^, ^22^, ^23^ Since the publication of the most recent treatment guidelines, two additional treatment options have been approved for LTP against HAE: garadacimab (Andembry^®^; SC; described below) and donidalorsen (Dawnzera^TM^; SC), a plasma prekallikrein‐directed antisense oligonucleotide.^24^, ^25^, ^26^

**TABLE 1:** Approved first‐line treatments recommended by current treatment guidelines for LTP for HAE.^2^, ^6^

Treatment	Mechanism of action	Key outcomes from clinical trials	Approved indication (FDA/EMA), dosing regimen, and method of administration
pdC1INH (IV; Cinryze^®^, Takeda)^6^, ^18^, ^20^, ^63^, ^64^	Inhibits plasma kallikrein, FXIIa, FXla, C1s, C1r, MASP‐1, MASP‐2, and plasmin	*Primary endpoint* − Mean monthly attack rate: ∼2.09^*^ − Reduction in mean monthly attack rate: NR *Secondary endpoint* − Mean monthly ODT injections: 1.57^†^	Patients aged ≥ 6 years with HAE (FDA/EMA): 500 IU (6–11 years) or 1,000 IU (≥ 12 years) IV every 3–4 days. Dose may be adjusted based on individual response
pdC1INH (SC; Berinert^®^ 2000/3000, HAEGARDA^®^, CSL Behring)^6^, ^19^, ^21^, ^64^, ^65^	Inhibits plasma kallikrein, FXIIa, FXla, C1s, C1r, MASP‐1, MASP‐2, and plasmin	*Primary endpoint* − Mean monthly attack rate: 0.52 − Reduction in mean monthly attack rate^‡^: 84% *Secondary endpoint* − Proportion of attack‐free patients: 40%	Patients aged ≥ 6 years with HAE (FDA); adults and adolescent patients with HAE‐C1INH (EMA): 60 IU/kg SC every 3–4 days
Lanadelumab (Takhzyro^®^, Takeda)^22^, ^66^, ^67^	Inhibits plasma kallikrein	*Primary endpoint* − Mean monthly attack rate: 0.26 (q2w), 0.53 (q4w) − Reduction in mean monthly attack rate^‡^: 87% (q2w), 73% (q4w) *Secondary endpoint* − Proportion of attack‐free patients: 44% (q2w), 31% (q4w)	Patients aged ≥ 12 years with HAE (FDA/EMA): 300 mg SC q2w/300 mg q4w^¶^ Patients aged ≥ 2 to < 12 years (EMA only): 150 mg q4w (10 to < 20 kg)^**^ or 150 mg q2w (20 to < 40 kg)^††^ or 300 mg q2w (≥ 40 kg)^‡‡^ Patients aged ≥ 2 to < 12 years (FDA only): 150 mg q4w (≥ 2 to < 6 years) 150 mg q2w (≥ 6 to < 12 years)^§§^
Berotralstat (Orladeyo^®^, BioCryst)^23^, ^68^, ^69^	Inhibits plasma kallikrein	*Primary endpoint* − Monthly attack rate: 1.31 (150 mg), 1.65 (110 mg) − Reduction in monthly attack rate^‡^: 44% (150 mg), 30% (110 mg) *Secondary endpoint* − Proportion of patients with a ≥ 90% reduction in attack rate: ≤ 23%^§^	Patients aged ≥ 12 years with HAE (FDA/EMA): 150 mg qd orally

*Abbr*.: C1r, complement component 1r; C1s, complement component 1s; EMA, European Medicines Agency; FDA, Food and Drug Administration; FXIa, activated factor XI; FXIIa, activated factor XII; HAE, hereditary angioedema; HAE‐C1INH, hereditary angioedema due to C1 inhibitor deficiency or dysfunction; IU, international unit; IV, intravenous; LTP, long‐term prophylaxis; MASP, mannose‐binding lectin‐associated serine protease; NR, not reported; ODT, on‐demand therapy; pdC1INH, plasma‐derived C1 inhibitor; q2w, every 2 weeks; q3w, every 3 weeks; q4w, every 4 weeks; qd, once daily; SC, subcutaneous

* Calculated from the mean of 6.26 attacks during the 12‐week treatment period

^†^ Calculated from the mean of 4.7 rescue infusions during a 12‐week treatment period

^‡^ Reduction versus placebo

^§^ Not significantly different from placebo

^¶^ May be considered if the disease is well controlled (e.g., patient is attack‐free) for > 6 months

** Dose can be increased to 150 mg q3w if insufficient control

^††^ Dose can be reduced to 150 mg q4w if stable

^‡‡^ Dose can be reduced to 300 mg q4w if stable

^§§^ Dose can be reduced to 150 mg q4w if stable.

## GARADACIMAB

### Mechanism of action

Targeting FXIIa, the principal initiator of the kallikrein–kinin system, represents an innovative treatment strategy for HAE.^16^, ^27^ Garadacimab is a first‐in‐class, fully human, immunoglobulin G4 anti‐FXIIa monoclonal antibody that inhibits FXIIa with high affinity, potency, and specificity, thus inhibiting the start of the bradykinin‐forming kallikrein–kinin system (Figure 2).^27^, ^28^, ^29^, ^30^

### Clinical development program and approval overview

Garadacimab is continuing to be evaluated for the LTP of HAE in a comprehensive clinical program in adult (aged > 17 years), adolescent (aged 12–17 years), and pediatric (aged 2–11 years) patients with HAE (Table 2).^28^, ^31^, ^32^, ^33^, ^34^ Based on the results observed with SC garadacimab (2 × 200 mg loading dose followed by 200 mg once monthly) in the pivotal phase III (VANGUARD) study, garadacimab has received regulatory approval from the *European Medicines Agency*, the *US Food and Drug Administration*, and regulatory bodies in Australia, Canada, Japan, Switzerland, the UAE, and the UK as LTP to prevent HAE attacks in adults and adolescents aged ≥ 12 years.^24^, ^25^, ^35^, ^36^, ^37^, ^38^, ^39^, ^40^, ^41^

**TABLE 2:** Overview of garadacimab clinical trials.

	Phase II^28^, ^31^	Pivotal phase III (VANGUARD)^32^	Phase III OLE^†^, ^33^	Phase III pediatric study^†^, ^34^
Study design	Multicenter, double‐blind, placebo‐controlled RCT with OLE	Double‐blind, placebo‐controlled RCT	OLE	Multicenter, open‐label, single‐arm
Eligible patients	HAE‐C1INH HAE‐FXII/‐PLG	HAE‐C1INH	Rollover patients from phase II or phase III studies, and newly enrolled patients	HAE‐C1INH
Number of patients	44^*^	64	161	20 (target)
Age, years	18–65	≥ 12	≥ 12	2–11
Treatment arms	TP1: IV loading dose of garadacimab 40 mg/100 mg/ 300 mg or placebo on day 1, followed by garadacimab 75 mg/200 mg/600 mg or placebo q1m SC	SC loading dose of garadacimab (2 × 200 mg doses) or volume‐matched placebo on day 1, followed by garadacimab 200 mg or placebo q1m SC	Garadacimab 200 mg q1m SC (newly enrolled patients received 2 × 200 mg SC loading dose as their first dose)	Dose A SC (aged 2–5 years) Dose B SC (aged 6–11 years)
	TP2: garadacimab 200 mg/600 mg q1m SC			
Trial duration	TP1: 13 weeks TP2: ≥ 44 weeks	6 months	≥ 12 months	12 months
Primary endpoint	Time‐normalized number of HAE attacks	Time‐normalized number of HAE attacks	TEAEs	TEAEs, PK

*Abbr*.: HAE, hereditary angioedema; HAE‐C1INH, HAE with C1‐esterase inhibitor deficiency or dysfunction; HAE‐FXII/‐PLG, hereditary angioedema with factor XII or plasminogen mutations; IV, intravenous; OLE, open‐label extension; PK, pharmacokinetics; q1m, every month; RCT, randomized controlled trial; SC, subcutaneous; TEAE, treatment‐emergent adverse event; TP, treatment period

* Including six patients with HAE‐XII/‐PLG; details of these patients are reported separately^51^

^†^ Trial in progress.

The efficacy and safety of garadacimab for LTP against HAE attacks was first evaluated in a phase II, randomized, dose‐finding study over two treatment periods of 13 weeks and ≥ 44 weeks (NCT03712228) (Table 2).^28^, ^31^

Following the phase II dose‐finding study, garadacimab 200 mg SC was selected for further investigation in a double‐blind, randomized, placebo‐controlled, pivotal phase III (VANGUARD) study (NCT04656418) (Table 2).^32^ In this study, 64 adult and adolescent patients with HAE‐C1INH were randomized 3:2 to either once‐monthly SC garadacimab 200 mg (n = 39) or placebo (n = 25) for a treatment duration of 6 months. The primary endpoint was the time‐normalized number of HAE attacks (monthly attack rate), and the secondary safety endpoints were adverse events (AEs) including AEs of special interest (AESIs) per protocol (abnormal bleeding events, thromboembolic events, severe hypersensitivity, and anaphylaxis), serious AEs (SAEs), and clinically significant abnormalities in laboratory assessments; concentrations of anti‐drug antibodies were also measured.^32^

The long‐term (≥ 12 months) efficacy and safety of garadacimab 200 mg SC once monthly in adult and adolescent patients are currently under evaluation in an ongoing phase III open‐label extension (OLE) study (NCT04739059) (Table 2).^33^ In total, 161 patients entered the OLE: 35 who were rolled over from treatment period 2 (TP2) of the phase II study, 57 from the pivotal phase III (VANGUARD) study, and 69 newly enrolled.^33^ The primary endpoint of the phase III OLE study is treatment‐emergent AEs (TEAEs) in patients with HAE‐C1INH. Secondary endpoints include long‐term efficacy measures (e.g., monthly attack rate, attack rate reductions versus run‐in, number of moderate and severe attacks) and safety measures (e.g., SAEs, deaths, TEAEs leading to discontinuation, and AESIs per protocol).^33^

Garadacimab is also being evaluated for 12 months in an ongoing multicenter, single‐arm, open‐label, phase III study in pediatric patients (aged 2–11 years) with HAE‐C1INH (NCT05819775) (Table 2).^34^ This study has a target enrollment of 20 patients and its primary endpoints include TEAEs and pharmacokinetics. Secondary efficacy endpoints include monthly attack rate.^34^

### Pharmacokinetics and pharmacodynamics of garadacimab

Data from adults and adolescents who received garadacimab in phase I, II, and III clinical studies of healthy volunteers and patients with HAE were used to develop population pharmacokinetics and pharmacodynamics models, and data from those included in the phase II and III studies were used to develop an exposure–response model to evaluate the rationale for garadacimab 200 mg once‐monthly dosing.^42^

Models that adequately characterized the pharmacokinetics and pharmacodynamics of garadacimab demonstrated that steady‐state exposure was reached as early as one week after the first administration of a loading dose (2 × 200 mg SC injections), with an elimination half‐life of 8.5 days.^42^ Exposure–response analysis demonstrated that garadacimab 200 mg SC once monthly led to 75% of patients reaching the target therapeutic threshold (90% reduction in relative risk of attack versus run‐in).^42^ These data support the use of garadacimab 200 mg SC once‐monthly dosing without the need for dose adjustment.^42^

### Efficacy of garadacimab

In the 6‐month pivotal phase III (VANGUARD) study, the monthly attack rate (95% confidence intervals [CIs]) was similar for patients in both treatment arms during run‐in (3.1 [2.4–3.7] for the garadacimab 200 mg SC once monthly arm and 2.5 [2.1–2.9] for the placebo arm).^32^ The mean monthly attack rate (95% CIs) was lower with garadacimab (0.27 [0.05–0.49]) than with placebo (2.01 [1.44–2.57]), corresponding to a statistically significant mean reduction of 87% (*p* < 0.0001) (Table 3).^32^ The median monthly attack rate (interquartile range [IQR]) was 0 (0.0–0.31) with garadacimab versus 1.35 (1.00–3.20) with placebo, although percentage reduction versus run‐in was not reported. Additionally, 62% of patients receiving garadacimab remained attack‐free through the 6‐month study period versus none of the patients receiving placebo (*p* < 0.0001).^32^

**TABLE 3:** Key efficacy data from the pivotal phase III (VANGUARD) and phase III OLE studies.

Parameter	Pivotal phase III (VANGUARD) study^32^		Phase III OLE study (N = 161)^33^	
	*Garadacimab 200 mg (n = 39)*	*Placebo (n = 25)*	*Garadacimab*	*Run‐in*
Treatment period, months	6		Median (IQR): 13.8 (11.9–16.3)	
Mean monthly attack rate (95% CIs)	0.27 (0.05–0.49)	2.01 (1.44–2.57)	0.16 (0.10–0.22)	3.57 (3.20–3.95)
Mean percentage difference (95% CIs) in number of attacks per month versus placebo/run‐in	−87 (–96 to –58)		−95 (–97 to –93)	
Median monthly attack rate (IQR)	0 (0.00–0.31)	1.35 (1.00–3.20)	0 (0.00–0.13)	2.85 (1.90–4.35)
Median percentage difference (IQR) in number of attacks versus run‐in	–^*^		−100 (–100 to –96)	
Number (%) of attack‐free patients^†^	24 (62)	0	96 (60)	–
Mean monthly attack rate requiring ODT (95% CIs)	0.23 (0.02–0.45)	1.86 (1.26–2.46)	0.14 (0.09–0.20)	2.98 (2.57–3.40)
Mean monthly number of moderate/severe attacks (95% CIs)	0.13 (0.03–0.22)	1.35 (0.86–1.84)	0.11 (0.07–0.15)	2.59 (2.26–2.92)

*Abbr*.: CI, confidence interval; HAE, hereditary angioedema; IQR, interquartile range; ODT, on‐demand treatment; OLE, open‐label extension

* Median percentage difference (IQR) in number of attacks versus run‐in was not reported in the pivotal phase III (VANGUARD) study^32^

^†^ Attack‐free patients are those that had 100% reduction in HAE attack rate.

The findings from the first interim analysis of the ongoing phase III OLE study (data cut‐off: February 2023) were consistent with those of the 6‐month pivotal phase III (VANGUARD) study, demonstrating durable efficacy and sustained protection from HAE attacks with garadacimab 200 mg SC once monthly over a median exposure of 13.8 months (Table 3).^32^, ^33^ The mean monthly attack rate (95% CIs) was 0.16 (0.10–0.22) during garadacimab treatment versus 3.57 (3.20–3.95) during run‐in, corresponding to a mean reduction of 95%.^33^ The median monthly attack rate (IQR) was 0 (0.0–0.13) during garadacimab treatment versus 2.85 (1.90–4.35) during run‐in, corresponding to a median reduction of 100%.^33^ Consistent with previous data, 60% of patients receiving garadacimab were attack‐free up to data cut‐off.^33^ Additionally, 21/22 (95%) patients who received garadacimab in the rollover group who remained attack‐free throughout the pivotal phase III (VANGUARD) study maintained attack‐free status across the entire OLE, over a median 14.0 months of garadacimab exposure.^33^

Results from a post hoc integrated efficacy analysis comprising the 6‐month pivotal phase III (VANGUARD) and long‐term OLE studies (median exposure: 1.2 years) have further corroborated the durable efficacy and sustained protection observed with garadacimab 200 mg SC once monthly.^43^ The mean monthly attack rate was 0.17 (standard deviation 0.40) during garadacimab treatment versus 3.55 (2.40) during run‐in, corresponding to a mean reduction of 94%.^43^

Garadacimab shows an early onset of protection against HAE attacks, as determined in a post hoc analysis of the pivotal phase III (VANGUARD) study. As early as one week after first administration, garadacimab reduced the mean monthly attack rate versus placebo.^44^ At week 1, the mean monthly attack rate (95% CIs) in the garadacimab group was 0.11 (–0.11 to 0.34) versus 3.07 (2.41–3.73) at run‐in compared with 1.81 (0.74–2.88) versus 2.52 (2.13–2.91) at run‐in in the placebo group.^44^ Achievement of garadacimab steady‐state exposures after the first administration of a loading dose may underlie the observed early onset of protection against HAE attacks.^42^ The mean monthly attack rate remained lower with garadacimab versus run‐in and versus placebo through month 6.^44^

Furthermore, garadacimab was associated with statistically significant improvements in all efficacy outcomes assessed versus lanadelumab 300 mg administered every 4 weeks in a matching‐adjusted indirect comparison study. Data from the garadacimab phase II and pivotal phase III studies were compared with published phase III summary‐level data from the lanadelumab HELP trial (NCT02586805). Garadacimab 200 mg SC once monthly reduced the overall HAE attack rate (rate ratio [RR] 0.29 [95% CI 0.13–0.63]), the rate of attacks requiring on‐demand treatment (RR 0.29 [95% CI 0.13–0.66]), the rate of moderate and/or severe HAE attacks (RR 0.15 [95% CI 0.05–0.49]), and increased the proportion of attack‐free patients (hazard ratio 3.25 [95% CI 1.45–7.29]) versus lanadelumab 300 mg administered every 4 weeks (*p* < 0.05 for each measure).^45^ Garadacimab also significantly reduced the rate of moderate and/or severe HAE attacks (RR 0.25 [95% CI 0.07–0.84]; *p* < 0.05) versus lanadelumab 300 mg every 2 weeks, but did not significantly reduce overall HAE attack rate.^45^ Consistent with this finding, garadacimab 200 mg once monthly reduced HAE attack rate versus C1INH SC, berotralstat, and lanadelumab 300 mg every 4 weeks in a network meta‐analysis (NMA), but did not significantly reduce attack rate versus lanadelumab 300 mg every 2 weeks.^45^ This NMA concluded that garadacimab had the highest probability of being the most effective treatment for reduction in HAE attacks (73%), with lanadelumab every 2 weeks ranking second (26%).^45^ Garadacimab was consistently associated with the highest probability of achieving efficacy endpoints, including increased proportion of attack‐free patients (55% versus 28% with lanadelumab every 2 weeks), reduction in HAE attacks requiring on‐demand treatment (53% versus 35% with C1INH SC), and reduction in moderate and/or severe HAE attacks (98% versus 1% with C1INH SC).^45^

### Safety of garadacimab

During the 6‐month pivotal phase III (VANGUARD) study, the proportion of patients experiencing at least one TEAE was comparable across treatment arms (64% of patients treated with garadacimab [75 TEAEs total] versus 60% of patients receiving placebo [54 TEAEs total]).^32^ The proportion of TEAEs considered related to study treatment was comparable between the garadacimab (10%) and placebo arms (12%) (Table 4).^32^ The majority of TEAEs resolved before the end of the study in both treatment groups.^32^

**TABLE 4:** Key safety data from the pivotal phase III (VANGUARD) and phase III OLE studies.

Endpoint, n (%)	Pivotal phase III (VANGUARD) study^32^		Phase III OLE study (N = 161)^33^
	*Garadacimab 200 mg (n = 39)*	*Placebo (n = 25)*	
Patients with ≥ 1 TEAE	25 (64)	15 (60)	133 (84)^*^
TEAEs related to study treatment	4 (10)	3 (12)	21 (13)
TEAEs leading to death	0	0	0
TEAEs leading to study discontinuation	0	0	1 (1)^‡^
TEAEs by severity			
Mild	NS	NS	101 (63)
Moderate	NS	NS	81 (50)
Severe	NS	NS	9 (6)
Serious TEAEs	1 (3)^†^	0	3 (2)^§^
Serious TEAEs related to study treatment			
AESIs per protocol^¶^	0	0	0
Most common TEAE (≥ 5% of patients)			
URTI	4 (10)	2 (8)	9 (6)
Nasopharyngitis	3 (8)	1 (4)	27 (17)
Headache	3 (8)	4 (16)	10 (6)
ISR^**^	2 (5)	3 (12)^††^	19 (12)
COVID‐19	–	–	58 (36)
Influenza	–	–	11 (7)

*Abbr*.: AESI, adverse event of special interest; HAE‐C1INH, hereditary angioedema with C1‐esterase inhibitor deficiency or dysfunction; ISR, injection‐site reaction; NS, not stated; OLE, open‐label extension; SAE, serious adverse event; TEAE, treatment‐emergent adverse event; URTI, upper respiratory tract infection.

Data are number of patients who had events, presented as n (%).

* Primary endpoints assessed based on the primary analysis safety set of patients with HAE‐C1INH (N = 159)

^†^ One severe SAE (laryngeal attack) was assessed as not related to study treatment: the patient made a full recovery and was kept under hospital observation overnight

^‡^ A garadacimab‐related TEAE occurring after 4 months of treatment (injection‐site irritation in the abdomen of moderate severity, within 24 h after injection; recovered/resolved after 13 days)

^§^ Three SAEs were reported in OLE (COVID‐19, n = 2; abdominal HAE attack, n = 1); none were related to garadacimab

^¶^ AESIs per protocol include abnormal bleeding events, thromboembolic events, severe hypersensitivity, and anaphylaxis

** In the pivotal phase III (VANGUARD) study all ISRs were mild; in the phase III OLE study most ISR events (43) were mild (41, 95%) and the remaining two were moderate in severity

^††^ One vaccination‐site reaction was recorded as an ISR.

In the phase III OLE study, the safety profile of garadacimab was generally consistent with that in the pivotal phase III (VANGUARD) study. In the overall OLE study population, 135/161 (84%) patients experienced at least one TEAE, with 21/161 (13%) patients experiencing at least one TEAE related to garadacimab (Table 4).^33^

Throughout the entire clinical development program, no deaths were reported.^28^, ^32^, ^33^, ^43^ One TEAE led to study discontinuation (a moderate garadacimab‐related injection‐site reaction [ISR] that occurred after month 4 in the phase III OLE study).^33^

TEAEs experienced by patients treated with garadacimab have been mostly mild or moderate in nature.^28^, ^31^, ^33^ A limited number of SAEs have been reported during the garadacimab clinical development program: in the pivotal phase III (VANGUARD) study, one patient in the garadacimab arm experienced a laryngeal attack, which required hospital observation overnight but from which the patient made a full recovery; in the phase III OLE study, three SAEs were reported (COVID‐19, n = 2; abdominal HAE attack, n = 1). None of the SAEs were considered by the investigators to be related to garadacimab.^32^, ^33^

There were no treatment‐related AESIs per protocol (abnormal bleeding, thromboembolic events, severe hypersensitivity, and anaphylaxis) in the 6‐month pivotal phase III (VANGUARD) study, the phase III OLE study (median exposure 13.8 months), or TP2 of the phase II study (approximately 1.7 years’ exposure).^31^, ^32^, ^33^ During TP2 of the phase II study, one case of mild epistaxis was observed in a patient treated with garadacimab 600 mg once monthly; this was unrelated to garadacimab and required no treatment.^31^

The most common TEAEs (≥5% of patients) reported in the pivotal phase III (VANGUARD) study included upper respiratory tract infections (URTIs), nasopharyngitis, headaches, and ISRs; in the phase III OLE study, the most commonly reported TEAEs included COVID‐19, nasopharyngitis, ISRs, influenza, headaches, and URTIs.^32^, ^33^

Medication‐related drug interactions were not detected in a pharmacokinetics model based on pooled data from phase I, phase II, and phase III studies for those who received concomitant analgesic, antihistamine, anti‐inflammatory, or antibacterial medications alongside garadacimab. Consistent with the overall study populations, most (91.9%) TEAEs were unrelated to garadacimab treatment, and 97.6% were mild or moderate in severity. All garadacimab‐related TEAEs were mild or moderate in severity, and ISRs and headaches were the most commonly reported events.^46^ No abnormal bleeding events were reported in patients who received garadacimab with concomitant anticoagulant or antiplatelet therapy in the pivotal phase III (VANGUARD) and phase III OLE studies, and garadacimab had no impact on hemostasis across the HAE clinical program.^47^

### Impact of garadacimab on HRQoL and patient‐reported outcomes

In the pivotal phase III (VANGUARD) study, clinically meaningful improvements (≥ 6 points) were observed in *Angioedema Quality of Life Questionnaire* (AE‐QoL) total score as early as 31 days after the first administration of garadacimab (2 × 200 mg loading dose), with a 23.7‐point reduction from the run‐in period. This improvement was sustained through to day 182 with a 26.5‐point reduction. For patients receiving placebo, there was no improvement in AE‐QoL total score of ≥ 6 points at any time during the study.^32^

The improvements in AE‐QoL score associated with garadacimab were sustained in the phase III OLE study, where 82% of evaluable patients previously exposed to garadacimab had either stable or further clinically meaningful improvements from the final AE‐QoL score in the previous study. Meanwhile, 92% of evaluable patients who were garadacimab‐naïve had clinically meaningful improvements in AE‐QoL total score from baseline.^48^ In both the pivotal phase III (VANGUARD) and phase III OLE studies, patients who remained attack‐free had the greatest improvements in AE‐QoL score and the highest treatment satisfaction.^49^

Moreover, in the pivotal phase III (VANGUARD) study, a higher proportion of patients treated with garadacimab (82%) rated the treatment as “good” or “excellent,” as per the *Subject's Global Assessment of Response to Therapy*, compared with patients who received placebo (33%).^32^ The same trend was observed through month 12 in the phase III OLE study, where improvements in patient rating of response with garadacimab were sustained (93% rated response to treatment as “good” or “excellent”).^33^

Various other questionnaire tools, such as the *Work Productivity and Activity Impairment questionnaire* and the *Treatment Satisfaction Questionnaire for Medication version II*, have shown garadacimab to improve patient‐reported outcomes.^31^, ^48^

### Garadacimab in adolescent and pediatric patients

An adolescent subgroup analysis of the pivotal phase III (VANGUARD) and phase III OLE study (data cut‐off: February 13, 2023; median exposure: 16.3 months) demonstrated that garadacimab reduced the mean monthly attack rate versus run‐in within the adolescent subgroup (12–17 years of age).^50^ In the pivotal phase III (VANGUARD) study (n = 6), the reduction in mean monthly attack rate was presented for four individual patients treated with garadacimab (–5.8%, 92.3%, 100%, 100%) and two patients treated with placebo (–12.2%, 90.8%) versus run‐in. In the OLE study (n = 10), the reduction in mean monthly attack rate versus run‐in ranged from 68.2% to 100% with four patients becoming attack‐free (100% reduction). Garadacimab was well tolerated in adolescent patients, with no deaths, discontinuations, SAEs, AESIs per protocol, or garadacimab‐related TEAEs.^50^

To extend the evidence base for garadacimab in HAE across all ages, garadacimab is being evaluated in an ongoing phase III pediatric study expected to complete in 2026; further data will become available in due course.^34^

### Garadacimab in patients with HAE‐nC1INH

Patients with HAE‐nC1INH were included in the phase II and phase III OLE studies of garadacimab.^51^ Among the six patients with confirmed HAE due to FXII mutation (HAE‐FXII; n = 3) or due to plasminogen mutation (HAE‐PLG; n = 3) who received open‐label garadacimab 600 mg SC in treatment period 1 (TP1) of the phase II study, two patients with HAE‐FXII rolled over to TP2 (garadacimab 600 mg SC) and later the phase III OLE in which they received garadacimab 200 mg SC. The other four patients discontinued the study during (n = 1) or after TP1 (n = 3) citing lack of, or inconsistent, efficacy. Two patients with HAE‐FXII experienced a reduction in mean monthly attack rate > 88% versus run‐in in the phase II study and were attack‐free in the phase III OLE, whereas the third patient with HAE‐FXII experienced a 19% decrease in mean monthly HAE attack rate. During the phase II study, two patients with HAE‐PLG experienced an increase in mean monthly attack rate (198% and 119%, respectively), and the third patient with HAE‐PLG experienced a 45% decrease in monthly attack rate. Garadacimab was well tolerated in all six patients with HAE‐nC1INH. In the phase II study, four of the six patients (HAE‐FXII, n = 2; HAE‐PLG, n = 2) experienced TEAEs during TP1, including one mild garadacimab‐related ISR and one unrelated SAE of a severe HAE attack. The two patients with HAE‐FXII who continued the study experienced 13 mild/moderate TEAEs during TP2 of the phase II study, and two mild TEAEs during the phase III OLE.^51^

It should be noted that there are limited data regarding the use of garadacimab in patients with HAE‐nC1INH. Patients with HAE‐nC1INH represent a diverse population with a high unmet therapeutic need. Current treatment strategies for patients with HAE‐nC1INH are based on evidence generated primarily in patients with HAE‐C1INH.^51^ Additionally, some subcategories of HAE‐nC1INH may not respond to treatment with garadacimab due to an alternative, FXII‐independent, pathway of bradykinin production. It is recommended to perform genetic testing according to the current HAE guidelines, if available, and to discontinue garadacimab treatment if clinical response is not observed within 3 months.^25^

## GARADACIMAB IN THE CURRENT LANDSCAPE

The ultimate goal of HAE treatment as per the WAO/EAACI guidelines is to achieve total disease control and to normalize patients’ lives.^2^ Normalization of life encompasses attack rate reduction (as stipulated by the WAO/EAACI guidelines) and amelioration of disease burden.^2^, ^3^, ^6^, ^52^, ^53^

A considerable subset of patients with HAE‐C1INH continue to experience insufficient disease control with currently available prophylactic strategies;^54^, ^58^, ^59^ therefore, novel LTP treatment options are needed to help patients get closer to achieving total disease control.

Garadacimab has a mechanism of action that differs from those of other existing LTP treatments. It inhibits FXIIa of the kallikrein–kinin system, which, according to a recent mechanistic model, is a more effective treatment approach for the prevention of bradykinin generation than the inhibition of downstream enzymes.^27^, ^60^ Early studies in murine models also demonstrated that 3F7, the parental antibody of garadacimab, exhibits greater inhibition of bradykinin generation when compared with other downstream inhibitors, such as bradykinin B2 receptor inhibitor and active plasma kallikrein blockers.^27^, ^29^ The therapeutic potential for garadacimab in the current HAE landscape is of great interest for physicians and patients. Thus far, the clinical program has demonstrated garadacimab to have durable efficacy, with an early onset of protection from HAE attacks that has been sustained for approximately 2 years across the phase II and III clinical trials, offering patients the potential to live disease‐free for extended periods of time.^28^, ^31^, ^32^, ^33^, ^43^, ^44^ Further, a high proportion of patients treated with garadacimab were attack‐free throughout the pivotal phase III and OLE studies, showcasing its potential to help bring patients closer to the goal of complete disease control, per WAO/EAACI guidelines.^32^, ^33^, ^49^ In addition, the efficacy demonstrated with garadacimab translated into sustained and clinically meaningful improvement in HRQoL within 1 month of treatment, facilitating normalization of life in a large number of patients, particularly those who remained attack‐free.^32^, ^48^, ^49^ Garadacimab has also demonstrated a favorable long‐term safety profile, with mostly mild/moderate TEAEs, low rates of ISRs, and no AESIs per protocol.^31^, ^32^, ^33^ These safety and efficacy data remain consistent in the adolescent subgroup population, and further investigations into pediatric populations are anticipated, which will extend the data on garadacimab across all age groups.^34^, ^50^

Uncontrolled HAE often necessitates the need for emergency department visits, and patients with insufficient disease control experience high levels of anxiety and depression in anticipation of their next attack.^3^, ^54^ Currently available therapies require frequent dosing, up to twice weekly, and patients with HAE may adjust their daily routines to accommodate their dosing regimen.^2^, ^61^, ^62^ In a 2020 US survey, 20% of patients reported that administration time of prophylactic treatment negatively impacted their daily life, with some reporting skipped medications due to treatment inconvenience.^2^ Additionally, improved LTP effectiveness, tolerability, and administration routes and schedules can positively impact patient HRQoL. Novel LTP therapies can provide convenient dosing to improve adherence and patient HRQoL.^55^, ^56^, ^57^, ^61^, ^62^ In addition to its efficacy and safety profile, garadacimab 200 mg SC once monthly has the potential to improve convenience of administration, patient compliance, and HRQoL, owing to its dosing schedule and method of administration, minimizing disruption to the daily lives of patients.^28^, ^32^, ^33^, ^48^

## FUTURE PERSPECTIVES

Based on clinical evidence to date, garadacimab demonstrates potential as a new treatment option in the HAE landscape, offering patients the opportunity to get as close as possible to total disease control and normalization of life as per the treatment goals set out in current clinical guidelines. Long‐term safety and efficacy data are still being collected in the ongoing phase III OLE, and the suitability of garadacimab for pediatric patients is being evaluated.^33^, ^34^ This is especially important and a significant unmet need, as pediatric patients have limited LTP treatment options with only lanadelumab approved for those aged ≥ 2 years and pdC1INH approved for those aged ≥ 6 years.^6^, ^18^, ^19^, ^20^, ^22^

## CONCLUSIONS

Garadacimab, administered SC (2 × 200 mg loading dose followed by 200 mg once monthly), is a novel, high‐affinity, fully human immunoglobulin G4 recombinant monoclonal antibody targeting FXIIa in the kallikrein–kinin system. Garadacimab is approved as LTP for the routine prevention of HAE attacks in multiple countries. Furthermore, garadacimab demonstrated early onset and durable efficacy with sustained protection against HAE attacks with a favorable long‐term safety profile across the clinical program. Garadacimab could potentially help to bring patients with HAE closer to achieving the guideline‐recommended goals of disease control and normalization of life.

## FUNDING

Funding for medical writing support was provided by CSL Behring, LLC.

## CONFLICT OF INTEREST STATEMENT

E.A.‐P. has received personal and/or institutional honoraria as a speaker/advisor for and/or grant/clinical trial investigator support from Astria Therapeutics, BioCryst, BioMarin, CSL Behring, Intellia Therapeutics, KalVista, Otsuka, Pharming, Pharvaris, and Takeda/Shire outside the submitted work. R.T. received personal fees from Takeda and CSL Behring and took part in clinical trials initiated by Takeda and CSL Behring outside the submitted work. P.S. received honoraria, research funding, and travel grants from and/or has served as a consultant for and/or has participated in advisory boards for BioCryst, CSL Behring, KalVista, Octapharma, Pharming, Pharvaris, Shire, and Takeda outside the submitted work. I.M.S. received research funding support and/or speaker/consultant fees from BioCryst, CSL Behring, KalVista Pharmaceuticals, Octapharma, Pharming, Pharvaris, and Takeda/Shire outside the submitted work. T.L. is an employee of CSL Behring. M.M. has received honoraria as a speaker/advisor from Astria, BioCryst, CSL Behring, Intellia, KalVista, Octapharma, Otsuka, Pharvaris, and Takeda/Shire outside the submitted work.
